# Risk of Mortality among Patients with Gastrointestinal Bleeding with Early and Late Treatment with Tranexamic Acid: A Population-Based Cohort Study

**DOI:** 10.3390/jcm11061741

**Published:** 2022-03-21

**Authors:** Ke-Hsin Ting, Bei-Hao Shiu, Shun-Fa Yang, Pei-Lun Liao, Jing-Yang Huang, Yin-Yang Chen, Chao-Bin Yeh

**Affiliations:** 1 Division of Cardiology, Department of Internal Medicine, Changhua Christian Hospital, Yunlin Branch, Yunlin 648, Taiwan; patrickting3@kimo.com; 2 Institute of Medicine, Chung Shan Medical University, Taichung 402, Taiwan; shiubeihao@gmail.com (B.-H.S.); ysf@csmu.edu.tw (S.-F.Y.); liaopeilun0410@gmail.com (P.-L.L.); wchinyang@gmail.com (J.-Y.H.); 3 Department of Surgery, Chung Shan Medical University Hospital, Taichung 402, Taiwan; 4 School of Medicine, Chung Shan Medical University, Taichung 402, Taiwan; 5 Department of Medical Research, Chung Shan Medical University Hospital, Taichung 402, Taiwan; 6 Department of Emergency Medicine, School of Medicine, Chung Shan Medical University, Taichung 402, Taiwan; 7 Department of Emergency Medicine, Chung Shan Medical University Hospital, Taichung 402, Taiwan

**Keywords:** tranexamic acid, gastrointestinal bleeding, mortality, thromboembolic events

## Abstract

Tranexamic acid (TXA) is an antifibrinolytic pharmacological agent, but its use in gastrointestinal bleeding remains contentious. Moreover, studies on the timing of TXA administration are limited. We examined whether early TXA administration reduced the risk of mortality in patients with gastrointestinal bleeding in a Taiwanese population. We used the National Health Insurance Research Database to identify patients diagnosed with gastrointestinal bleeding with early and late TXA treatment. We defined early treatment as initial TXA treatment in an emergency department and late treatment as initial TXA treatment after hospitalization. Mortality within 52 weeks was the primary outcome. A multivariable analysis using a multiple Cox regression model was applied for data analysis. Propensity score matching (PSM) was performed to reduce the potential for bias caused by measured confounding variables. Of the 52,949 selected patients with gastrointestinal bleeding, 5127 were assigned to either an early or late TXA treatment group after PSM. The incidence of mortality was significantly decreased during the first and fourth weeks (adjusted HR (aHR): 0.65, 95% CI: 0.56–0.75). A Kaplan–Meier curve revealed a significant decrease in cumulative incidence of mortality in the early TXA treatment group (log-rank test: *p* < 0.0001). Multiple Cox regression analysis revealed significantly lower mortality in the early TXA treatment group compared with the late treatment group (aHR: 0.64, 95% CI: 0.57–0.73). Thromboembolic events were not significantly associated with early or late TXA treatment (aHR: 1.03, 95% CI: 0.94–1.12). A Kaplan–Meier curve also revealed no significant difference in either venous or arterial events (log-rank test: *p* = 0.3654 and 0.0975, respectively). In conclusion, early TXA treatment was associated with a reduced risk of mortality in patients with gastrointestinal bleeding compared with late treatment, without an increase in thromboembolic events. The risk of rebleeding and need for urgent endoscopic intervention require further randomized clinical trials.

## 1. Introduction

Acute gastrointestinal bleeding is a common cause of morbidity and mortality worldwide [1] with a reported mortality rate of 2–10% [2,3]. The bleeding can arise from both the upper and lower gastrointestinal tracts, including from peptic ulcers, esophageal or gastric varices, diverticulitis, colitis, or malignancy in the gastrointestinal tract. Clinical symptoms of acute gastrointestinal bleeding typically include hematemesis, melaena, or hematochezia. The initial management of gastrointestinal bleeding in emergency departments includes triage, supportive management, blood transfusion, fluid resuscitation, and endoscopic therapy, depending on the severity and hemodynamic status of patients.

Tranexamic acid (TXA) is an antifibrinolytic agent that reversibly inhibits the conversion of plasminogen to plasmin, resulting in a reduction in fibrinolysis. First introduced for menorrhagia in 1968 [4], TXA has the ability to reduce postoperative hemorrhage [5,6], postpartum hemorrhage [7], and mortality in patients with traumatic hemorrhage [8]. The role of TXA in acute gastrointestinal bleeding remains under debate, without clear recommendations for its clinical use. 

However, studies have demonstrated its efficacy in reducing the mortality rate of patients with acute gastrointestinal bleeding [9,10]. A recent systemic review and meta-analysis including 13 randomized trials with a total of 2271 patients with acute gastrointestinal bleeding revealed that TXA significantly reduced the mortality rate (relative risk (RR) = 0.60; 95% CI, 0.45–0.80) and rates of continued bleeding (RR = 0.60; 95% CI, 0.43–0.84) [11]. In contrast, another randomized controlled trial (HALT-IT trial) revealed that TXA did not significantly reduce mortality in patients with gastrointestinal bleeding (RR = 0.99, 95% CI, 0.82–1.18) [12]. However, adverse venous thromboembolic events were higher in patients using TXA than in those not using it.

A possible confounding factor is the timing of TXA administration, which has rarely been considered in studies and could affect study outcomes. Hence, this nationwide cohort study aimed to identify whether early or late use of TXA reduced the mortality of patients with gastrointestinal bleeding in Taiwan. We hypothesized that patients with gastrointestinal bleeding receiving TXA early would have lower risk of mortality.

## 2. Materials and Methods

### 2.1. Study Design and Population

In this retrospective cohort study, we analyzed the administration of TXA for gastrointestinal bleeding and the risk of all-cause mortality. Taiwan adopted a National Health Insurance system in 1995, this system claims data as the National Health Insurance Research Database (NHIRD). The NHIRD provides real-world evidence for exploring the risk factors or effects of an intervention for specific diseases, and contains the insurance claims data of more than 99% of Taiwan’s population. We used NHIRD data from between January 2000 and December 2017 to evaluate the risk of all-cause mortality among patients with gastrointestinal bleeding who received early or late TXA treatment. This study was approved by the Institutional Review Board of the Chung Shan Medical University Hospital (approval number CS2-20036).

### 2.2. Study Population

We initially included 52,949 hospitalized patients who went to an emergency department for gastrointestinal bleeding, as defined by the following International Classification of Diseases, Ninth Revision, Clinical Modification (ICD-9-CM) codes: 530.1, 530.2, 530.7, 531.0, 531.4, 531.9, 532.0, 532.4, 532.9, and 578. In addition, the following ICD-10-CM codes were applied: K20.0, K20.8, K20.9, K21.0, K22.10, K22.11, K22.6, K25.0, K25.4, K25.9, K26.0, K26.4, K26.9, K92.0, K92.1, and K92.2 (Supplementary Table S1). All the selected patients had a subsequent hospitalization record within 1 day of emergency care. The exclusion criteria were as follows: (1) an index date before 2000 or after 2016 (*n* = 6670); (2) lack of TXA treatment (ATC code: B02AA02) during emergency treatment or admission (*n* = 27,436); (3) cancer diagnosis before the index date (*n* = 4151); and (4) death before the index date (*n* = 6). This study included 14,686 patients with gastrointestinal bleeding who had received TXA treatment; among these patients, 9513 received early treatment, defined as initial TXA treatment in an emergency department, and 5173 received late treatment, defined as initial TXA treatment after hospitalization.

### 2.3. Characteristics, Comorbidities, and Study Outcomes

We identified the baseline (within 180 days of the index date) demographic characteristics, such as age and sex, and the comorbidities and medication of each participant to evaluate their health status. Comorbidities included hypertension, diabetes mellitus, hyperlipidemia, kidney disease, chronic pulmonary diseases, liver disease, ischemic heart diseases, ischemic stroke, hemorrhagic stroke, atrial fibrillation, congestive heart failure, dementia, and peripheral vascular disease. Medications included proton-pump inhibitors, hemostatic agents, drugs for constipation, furosemide, metoclopramide, silicon, magnesium oxide, aspirin, clopidogrel or ticagrelor, and nonsteroidal anti-inflammatory drugs. 

The study’s primary outcome was all-cause mortality within 52 weeks of the index date. The secondary outcomes were thromboembolic events (deep-vein thrombosis, pulmonary embolism, acute myocardial infarction, hemorrhagic stroke, and ischemic stroke). All the patients in the study were followed up from the index date until their withdrawal from the National Health Insurance program, the occurrence of a study event, or 52 weeks after the index date.

### 2.4. Statistical Analysis

Statistical analysis was performed using SAS software version 9.4 (SAS Institute, Cary, NC, USA), and a *p* value of < 0.05 was considered significant. The propensity score was the odds of gastrointestinal bleeding according to demographic data, including birth year, sex, age (±1 year) on the index date, index year, comorbidities, and medication. To reduce potential confounding bias caused by measured factors, 1:1 propensity score matching (PSM) was performed using greedy nearest neighbor non-replacement matching with a caliper width of 0.1. The difference in covariates between the 2 study groups was evaluated using the absolute standardized difference (ASD), as an absolute ASD value of ASD < 0.1 indicated that the groups were balanced with their matched control.

Categorical data are presented as numbers and percentages, and the differences in categorical variables were compared using a chi-square test. The incidence rate with the corresponding CIs and crude hazard ratios (HRs) were calculated using Poisson regression. After the proportional hazards assumption was tested, a Cox proportional hazards model analysis was performed to estimate the HRs for mortality and 95% CIs. The cumulative probabilities of mortality were assessed using a Kaplan–Meier analysis, with statistical significance being determined using the results of a log-rank test.

## 3. Results

### 3.1. Characteristics of the Participants

**Figure 1:**
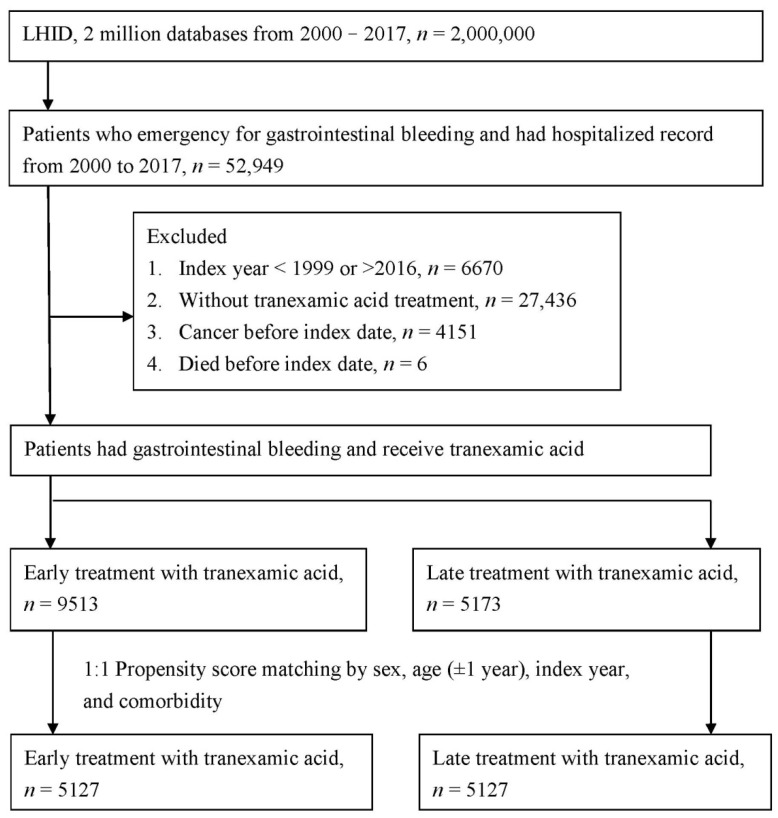
Study flowchart.

The study flowchart is presented in Figure 1. Of the patients with gastrointestinal bleeding administered TXA, 9513 received early treatment and 5173 received late treatment. In total, 67% of the patients were male and 32% were female, and more than 40% were aged ≥ 71 years. Before PSM, the statistically significant differences between the two groups were index year and medication (including hemostatic agents, drugs for constipation, furosemide, metoclopramide, and silicon). After PSM, the two groups were balanced, as indicated by the ASD of the covariates (Table 1).

### 3.2. The Risk of Mortality in the TXA Treatment Group

**Figure 2:**
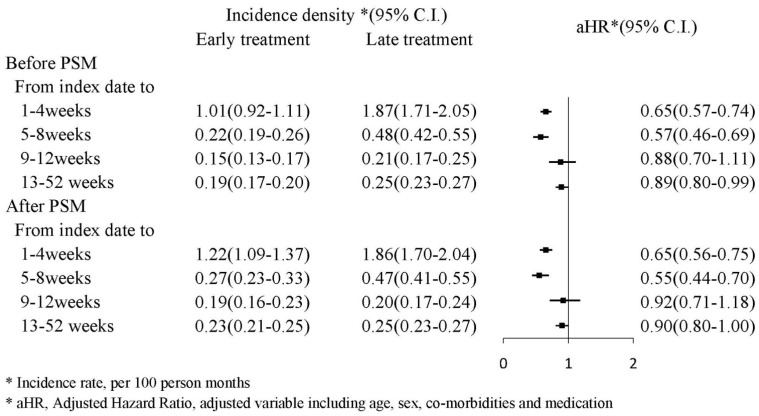
Incidence density of mortality.

**Figure 3:**
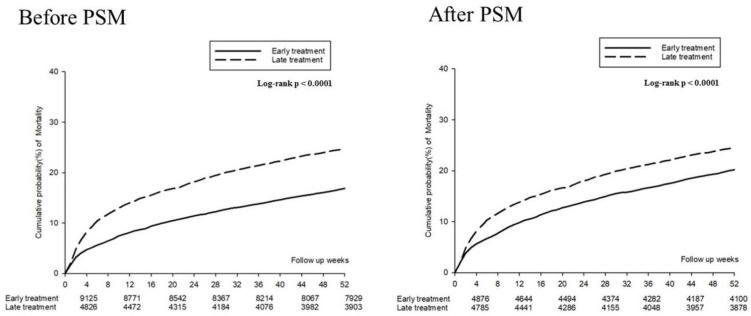
Kaplan–Meier curves for the 52 week mortality risk.

Figure 2 presents the mortality incidence density (per 100 person month), which were 1.22 (95% CI: 1.09–1.37) and 1.86 (95% CI: 1.70–2.04) in the PSM early and late treatment groups, respectively; the adjusted HR for early treatment was 0.65 (95% CI: 0.56–0.75) during the first and fourth weeks. During the 13th and 52nd weeks, the mortality incidence density (per 100 person month) was 0.23 (95% CI: 0.21–0.25) and 0.25 (95% CI: 0.23–0.27) in the early and late treatment groups, respectively; the adjusted HR (aHR) for early treatment was 0.90 (95% CI: 0.80–1.00). A Kaplan–Meier survival analysis revealed significantly lower cumulative incidence of mortality in the early treatment group (log-rank test: *p* < 0.0001; Figure 3).

The patients who received early TXA treatment had a significantly lower risk of mortality during the first and eighth weeks compared with those who received late treatment (aHR: 0.64, 95% CI: 0.57–0.73). Other significant risk factors of mortality were age; comorbid kidney disease, liver disease, and hemorrhagic stroke; and prescription for hemostatic agents, drugs for constipation, furosemide, and metoclopramide (Table 2).

**Figure 4:**
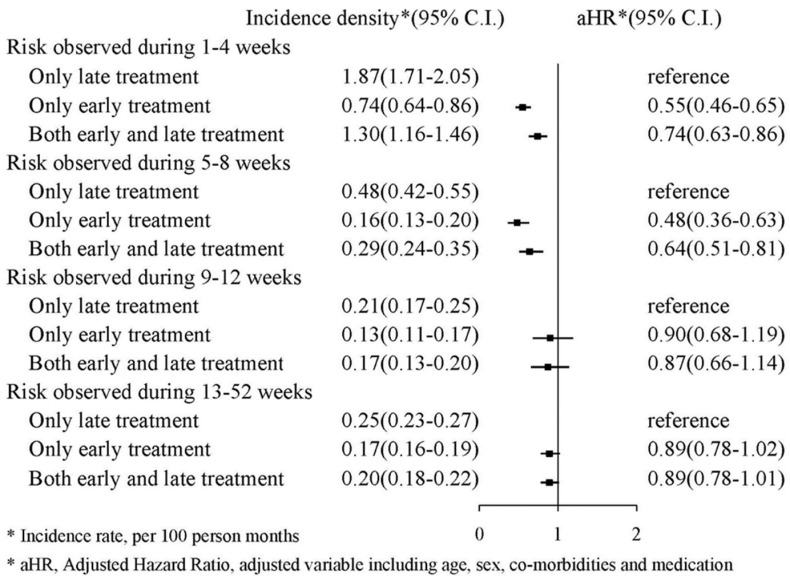
Incidence density of mortality.

We also classified the TXA treatment into three groups: (1) early TXA treatment in an emergency department; (2) late TXA treatment after hospitalization; and (3) both early and late TXA treatment. Figure 4 presents the risk of mortality in those who received early TXA treatment compared with those who only received late TXA treatment. Regarding risk during the first and fourth weeks, the aHR for early treatment was 0.55 (95% CI: 0.46–0.65), and the aHR for both early and late treatment was 0.74 (95% CI: 0.63–0.86). Regarding risk during the 13th and 52nd weeks, the aHR for early treatment was 0.89 (95% CI: 0.78–1.02), and the aHR for both early and late treatment was 0.89 (95% CI: 0.78–1.01).

### 3.3. Thromboembolic Events in the TXA Treatment

**Figure 5:**
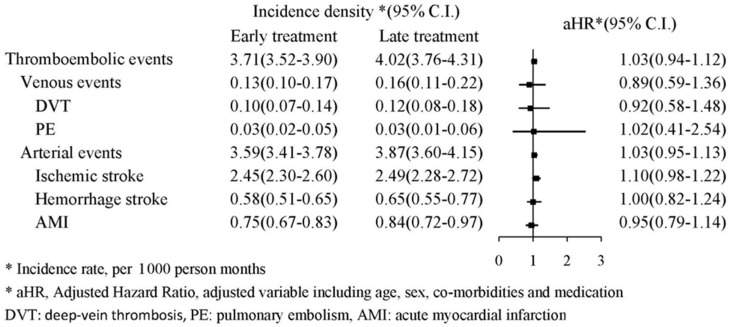
Flowchart of patient selection.

**Figure 6:**
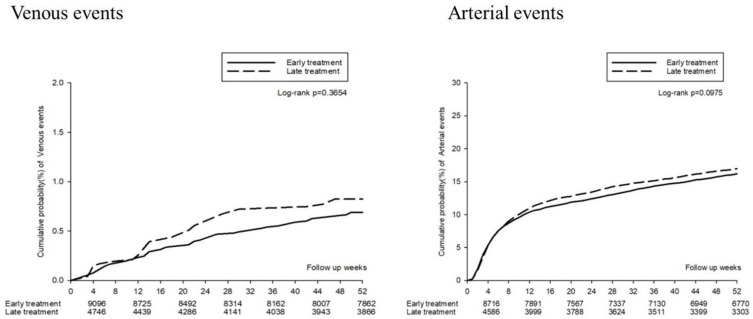
Kaplan–Meier curves for the 52 week thromboembolic events.

The thromboembolic events (including deep-vein thrombosis, pulmonary embolism, acute myocardial infarction, hemorrhagic stroke, and ischemic stroke) are presented in Figure 5. The forest plot analysis indicated no significant association between thromboembolic events and early or late TXA treatment (aHR: 1.03, 95% CI: 0.94–1.12). The Kaplan–Meier survival analysis also identified no significant increased cumulative probability of either venous or arterial events (log-rank: *p* = 0.3654 and 0.0975, respectively, Figure 6). 

## 4. Discussion

In this population-based trial, which included 10,254 patients with gastrointestinal bleeding, early TXA treatment was associated with 36% and 12% lower mortality for a follow-up period of 1 to 8 and 9 to 52 weeks, respectively. Moreover, early TXA treatment did not increase the risk of either venous or arterial thromboembolic events compared with late treatment.

TXA use for gastrointestinal bleeding has been evaluated in randomized clinical trials. In the HALT-IT trial, compared with the equivalent infusion of saline, TXA administration resulted in a lower risk of death caused by bleeding and rebleeding within 24 h, 5 days, and 28 days. However, an increased risk of venous thromboembolic events was observed [12]. Another randomized clinical trial, focusing on lower gastrointestinal bleeding, revealed that TXA had no benefits in relation to blood loss and clinical outcomes [13]. However, the results for TXA and outcomes for gastrointestinal bleeding have been inconsistent in recent systematic reviews and meta-analyses. Burke et al. performed a systemic review of 8 studies that included 12,994 patients with upper gastrointestinal bleeding. Although no effect on mortality was noted, the beneficial effect of TXA on lower rebleeding risk and a decreased need for surgery was observed [14]. Another systematic review and meta-analysis, including 13 relevant randomized clinical trials and a total of 2271 patients, demonstrated lower mortality and continued bleeding and less need for urgent endoscopic intervention [11].

Most of the aforementioned studies failed to consider a potential confounding factor: the timing of TXA administration. TXA can be prescribed for patients with gastrointestinal bleeding in an emergency department along with other initial treatments, or during hospitalization. For traumatic or postpartum hemorrhage, immediate TXA administration is recommended for improved survival [15]. Similarly, the timing of TXA treatment might affect its benefits for gastrointestinal bleeding, and trial results may be influenced by this confounding factor. Furthermore, current TXA treatment guidelines for gastrointestinal bleeding include no clear timings [16,17].

Hence, in our study design, we defined early treatment as TXA administered in an emergency department and late treatment as TXA administered after hospitalization. The results demonstrated a significant decrease in mortality rates for early TXA treatment in patients with gastrointestinal bleeding compared with late treatment for both short-term and long-term follow-up, which is compatible with the results of a systematic review and meta-analysis [11]. In addition to reducing mortality, early TXA administration in an emergency department was associated with a significant decrease in the need for urgent endoscopy in a randomized clinical trial exploring the effect of TXA on the urgent endoscopy rate for gastrointestinal bleeding [18].

The length of hospital stay is also a key clinical outcome. Our study demonstrated that early TXA treatment and both early and late treatment resulted in a shorter length of hospital stay compared with late treatment alone. Similar to our results, Miyamoto et al., who conducted a nationwide observational study in Japan, reported that TXA reduced the length of stay for patients with colonic diverticular bleeding [19]. Other retrospective studies have also revealed a decreased hospital stay following TXA administration for patients with vascular trauma [20] or intraoperative administration [21].

In addition to the common adverse effects of nausea, diarrhea, and stomach pain, the risk of thromboembolic events is a major adverse effect of TXA use in clinical practice [22,23]. The HALT-IT trial revealed an increase in venous thromboembolic events with TXA use, but primarily in patients with underlying liver diseases [12]. In contrast, the CRASH-3 trial demonstrated that TXA reduced the risk of death, with a similar risk of thromboembolic events compared with placebo groups [8]. Regarding the timing of TXA treatment, our study revealed a similar risk of thromboembolic events in early and late TXA treatment.

Our study also demonstrated that patients older than 71 years and those with liver and kidney disease all had a significantly increased risk of mortality from gastrointestinal bleeding. These results are consistent with those of a UK population-based study, which reported that older age was the most crucial prognostic factor for gastrointestinal bleeding, with a mortality rate 53 times higher for patients aged over 85 years. Liver and renal comorbidities were also associated with a 7.9 and 3.9 times higher mortality rate [24]. Another meta-analysis evaluated the relationship between kidney disease and outcomes of gastrointestinal bleeding, revealing a higher mortality in the chronic kidney disease group (odds ratio (OR): 1.786, 95% CI: 1.689–1.888, *p* < 0.001) and the end-stage renal disease group (OR: 2.530, 95% CI: 1.386–4.616, *p* = 0.002) [25].

Studies have demonstrated that TXA use leads to improved clinical outcomes for other hemorrhagic conditions, such as traumatic [8,26], major obstetric [27], postpartum [7], and surgical hemorrhage [5,28,29,30]. Studies have also considered the effect of TXA use on cerebral hemorrhage. A systematic review and meta-analysis including 14 randomized controlled trials with 4703 patients with cerebral hemorrhage demonstrated no improvement in mortality by day 90 in patients receiving TXA (OR: 0.99, 95% CI: 0.84–1.18, *p* = 0.95) [31]. However, the risk of rebleeding and hematoma expansion was reduced and thromboembolic events were not increased. Furthermore, Rowell et al. performed a double-blinded, randomized clinical trial of out-of-hospital TXA use within 2 h for neurological outcomes in patients with severe traumatic brain injury [32]. No significant difference in neurologic function at 6 months or mortality and progression of intracranial hemorrhage was observed.

This study has some limitations. First, data on personal behaviors, such as smoking and alcohol consumption, are not available in the NHIRD; such personal behaviors are potential confounders. However, to address these factors, we included related comorbidities and performed PSM. Second, the cause and location of the gastrointestinal bleeding, disease severity, endoscopic intervention and TXA dosage are not included in the NHIRD. Different hemorrhage severity, endoscopic intervention and TXA dosages are potential confounders of our results. Third, no control group of patients with gastrointestinal bleeding but without TXA treatment was included because our study evaluated the timing of TXA treatment. Finally, further randomized clinical trials with a sufficient sample size, rigorous patient selection, and controlled intervention are required.

## 5. Conclusions

In this Taiwan population-based study, early TXA treatment was associated with lower mortality without increased thromboembolic events compared with late treatment in patients with gastrointestinal bleeding. Future research is required to clarify the outcomes in terms of continued bleeding, rebleeding, blood transfusion, and the need for urgent endoscopic intervention.

## Figures and Tables

**Table 1:** Baseline characteristics among study groups.

	Before PSM			After PSM		
	Early Treatment	Late Treatment	ASD	Early Treatment	Late Treatment	ASD
*N*	9513	5173		5127	5127	
Index year			0.2488			0.0239
2000–2005	2064 (21.70%)	1596 (30.85%)		1516 (29.57%)	1551 (30.25%)	
2006–2010	2996 (31.49%)	1692 (32.71%)		1747 (34.07%)	1691 (32.98%)	
2011–2015	4453 (46.81%)	1885 (36.44%)		1864 (36.36%)	1885 (36.77%)	
Sex			0.0025			0.0042
Female	3060 (32.17%)	1658 (32.05%)		1633 (31.85%)	1643 (32.05%)	
Male	6453 (67.83%)	3515 (67.95%)		3494 (68.15%)	3484 (67.95%)	
Age			0.0405			0.0000
≤50	2443 (25.68%)	1277 (24.69%)		1289 (25.14%)	1268 (24.73%)	
51–70	3060 (32.17%)	1591 (30.76%)		1570 (30.62%)	1583 (30.88%)	
≥71	4010 (42.15%)	2305 (44.56%)		2268 (44.24%)	2276 (44.39%)	
CCI score			0.0885			0.0263
0	2225 (23.39%)	1009 (19.51%)		1043 (20.34%)	1006 (19.62%)	
1	2564 (26.95%)	1340 (25.90%)		1265 (24.67%)	1332 (25.98%)	
2	1848 (19.43%)	1101 (21.28%)		1056 (20.6%)	1088 (21.22%)	
≥3	2876 (30.23%)	1723 (33.31%)		1763 (34.39%)	1701 (33.18%)	
Co-morbidity						
Hypertension	4608 (48.44%)	2518 (48.68%)	0.0047	2494 (48.64%)	2495 (48.66%)	0.0004
Diabetes mellitus	2862 (30.09%)	1577 (30.49%)	0.0087	1565 (30.52%)	1564 (30.51%)	0.0004
Hyperlipidemia	1373 (14.43%)	652 (12.60%)	0.0535	637 (12.42%)	650 (12.68%)	0.0077
Kidney disease	1441 (15.15%)	965 (18.65%)	0.0937	940 (18.33%)	946 (18.45%)	0.0030
Chronic pulmonary diseases	1715 (18.03%)	1086 (20.99%)	0.0749	1023 (19.95%)	1065 (20.77%)	0.0203
Liver disease	3233 (33.99%)	1858 (35.92%)	0.0405	1880 (36.67%)	1840 (35.89%)	0.0162
Ischemic heart diseases	1598 (16.80%)	839 (16.22%)	0.0156	810 (15.80%)	834 (16.27%)	0.0128
Ischemic stroke	1045 (10.98%)	615 (11.89%)	0.0284	634 (12.37%)	606 (11.82%)	0.0167
Hemorrhage stroke	239 (2.51%)	170 (3.29%)	0.0461	161 (3.14%)	166 (3.24%)	0.0055
Atrial fibrillation	487 (5.12%)	258 (4.99%)	0.0060	252 (4.92%)	257 (5.01%)	0.0045
Congestive heart failure	1103 (11.59%)	667 (12.89%)	0.0396	647 (12.62%)	656 (12.80%)	0.0053
Dementia	631 (6.63%)	384 (7.42%)	0.0309	386 (7.53%)	380 (7.41%)	0.0045
Peripheral vascular disease	434 (4.56%)	213 (4.12%)	0.0218	198 (3.86%)	212 (4.13%)	0.0139
Medication						
Proton-pump inhibitors	8140 (85.57%)	4430 (85.64%)	0.0020	4403 (85.88%)	4389 (85.61%)	0.0078
Hemostatic	3237 (34.03%)	2085 (40.31%)	0.1302	2069 (40.35%)	2049 (39.96%)	0.0080
Drugs for constipation	5973 (62.79%)	3554 (68.70%)	0.1249	3489 (68.05%)	3508 (68.42%)	0.0080
Furosemide	3329 (34.99%)	2326 (44.96%)	0.2046	2291 (44.69%)	2283 (44.53%)	0.0031
Metoclopramide	4008 (42.13%)	2598 (50.22%)	0.1628	2564 (50.01%)	2553 (49.80%)	0.0043
Silicon	4157 (43.70%)	2539 (49.08%)	0.1081	2502 (48.80%)	2500 (48.76%)	0.0008
Magnesium oxide	1512 (15.89%)	910 (17.59%)	0.0455	887 (17.30%)	890 (17.36%)	0.0016
Aspirin	2833 (29.78%)	1744 (33.71%)	0.0846	1679 (32.75%)	1708 (33.31%)	0.0120
Clopidogrel/Ticagrelor	772 (8.12%)	398 (7.69%)	0.0156	392 (7.65%)	397 (7.74%)	0.0037
NSAIDs	6403 (67.31%)	3638 (70.33%)	0.0652	3603 (70.28%)	3598 (70.18%)	0.0021

ASD: absolute standardized difference, PSM: propensity score matching, CCI score: Charlson Comorbidity Index score.

**Table 2:** Multiple Cox regression to estimate the hazard ratio for the 52 week mortality risk.

Variable	aHR (95% CI)	
	1–8 Weeks	9–52 Weeks
Study group		
Early treatment	0.64 (0.57–0.73)	0.90 (0.80–1.00)
Late treatment	Reference	Reference
Index year		
2000–2005	Reference	Reference
2006–2010	1.23 (1.05–1.45)	0.98 (0.85–1.14)
2011–2015	1.12 (0.95–1.32)	1.04 (0.90–1.20)
Sex		
Female	Reference	Reference
Male	1.09 (0.95–1.25)	1.24 (1.10–1.40)
Age		
≤50	Reference	Reference
51–70	1.18 (0.96–1.45)	0.98 (0.81–1.18)
≥71	2.09 (1.70–2.57)	1.81 (1.51–2.18)
Co-morbidity (ref: non)		
Hypertension	0.74 (0.64–0.85)	0.94 (0.83–1.07)
Diabetes mellitus	1.07 (0.93–1.23)	1.24 (1.10–1.39)
Hyperlipidemia	0.76 (0.61–0.94)	0.73 (0.61–0.88)
Kidney disease	1.41 (1.21–1.63)	1.52 (1.34–1.73)
Chronic pulmonary diseases	1.09 (0.94–1.26)	1.22 (1.08–1.39)
Liver disease	1.32 (1.14–1.52)	1.48 (1.31–1.68)
Ischemic heart diseases	0.95 (0.79–1.13)	0.86 (0.73–1.00)
Ischemic stroke	1.01 (0.84–1.21)	1.05 (0.90–1.23)
Hemorrhage stroke	1.88 (1.45–2.44)	1.64 (1.27–2.11)
Atrial fibrillation	0.88 (0.68–1.13)	1.08 (0.88–1.33)
Congestive heart failure	1.18 (0.99–1.40)	1.11 (0.95–1.29)
Dementia	1.17 (0.96–1.42)	1.43 (1.21–1.69)
Peripheral vascular disease	1.23 (0.94–1.61)	1.09 (0.85–1.40)
Medication (ref: non)		
Proton-pump inhibitors	0.93 (0.75–1.14)	1.00 (0.84–1.20)
Hemostatic	1.95 (1.71–2.22)	1.27 (1.13–1.42)
Drugs for constipation	1.22 (1.02–1.45)	2.32 (1.93–2.79)
Furosemide	2.71 (2.32–3.16)	2.41 (2.12–2.74)
Metoclopramide	1.43 (1.25–1.64)	1.50 (1.33–1.69)
Silicon	0.85 (0.75–0.97)	0.97 (0.87–1.09)
magnesium oxide	0.95 (0.81–1.12)	0.92 (0.80–1.05)
Aspirin	1.11 (0.97–1.28)	1.06 (0.94–1.20)
Clopidogrel/Ticagrelor	1.09 (0.87–1.36)	1.27 (1.06–1.53)
NSAIDs	0.93 (0.75–1.14)	1.00 (0.84–1.20)

## Data Availability

Restrictions apply to the availability of these data. Data was obtained from National Health Insurance database and are available from the authors with the permission of National Health Insurance Administration of Taiwan.

## Supplementary Materials

The following supporting information can be downloaded at: https://www.mdpi.com/article/10.3390/jcm11061741/s1, Table S1: Definition of study diseases related to ICD-9-CM and ICD-10-CM.
