# Supplementary material for: Risk of Mortality among Patients with Gastrointestinal Bleeding with Early and Late Treatment with Tranexamic Acid: A Population-Based Cohort Study
Source: J Clin Med. 2022 Mar 21;11(6):1741. doi: 10.3390/jcm11061741 (PMC8951209; doi:10.3390/jcm11061741)
Supplement: Supplementary file 1 [file jcm-11-01741-s001.zip › jcm-1643799-supplementary.pdf]

Supplementary Table S1: Definition of study diseases related to ICD-9-CM and ICD-10-CM.

Disease	ICD-9-CM	ICD-10-CM
Gastrointestinal bleeding	530.1, 530.2, 530.7, 531.0, 531.4, 531.9, 532.0, 532.4, 532.9, 578	K20.0, K20.8, K20.9, K21.0, K22.10, K22.11, K22.6, K25.0, K25.4, K25.9, K26.0, K26.4, K26.9 ,K92.0, K92.1, K92.2
Hypertension	401.x-405.x	I10, I11.x, I13.x, I15.x, I16.x, I87.3x, I97.3x, O10.x, O11.x, O13.x, O16.x
Diabetes mellitus	250.x, 277.7	O24.4, E11.x, E13.x, E88.81
Hyperlipidemia	272.0, 272.1, 272.2, 272.4, 272.9	E78.0x, E78.1, E78.2, E78.3, E78.4x, E78.5, E78.70, E78.79, E78.89, E78.9
Kidney disease	403.x, 404.x, 582.x, 583.0–583.7, 585.x, 586.x, 588.x, V42.0, V45.1, V56.x	E08.2x, E09.2x, E11.2x, E13.2x, I12.x, I13.1, N03.x, N04.x, N11.x, N18.x, O10.2x, O10.3x, Z49.31, I95.3, T82.43XA, E85.3, R88.0, T82.4x
Chronic pulmonary diseases	416.8, 416.9, 490.x–505.x, 506.4, 508.1, 508.8	J41.x, J42, J43.x, J44.x, J47.x
Liver disease	070.22, 070.23, 070.32, 070.33, 070.44, 070.54, 070.6, 070.9, 456.0–456.2, 570.x, 571.x, 572.2–572.8, 573.3, 573.4, 573.8, 573.9, V42.7	K72.x-K77, T86.43, T86.49, Z94.4
Ischemic heart diseases	410.x, ,412.x, 414.0, 414.0x, 414.2, 414.3, 414.4, 414.8, 414.9	I20.x-I25.x
Ischemic stroke	433-435	I63, I65, I66, I67.84, G45, G46
Hemorrhagic stroke	430-432,	I60, I61, I62
Atrial fibrillation	427.3	I48

Congestive heart failure	398.91, 402.01, 402.11, 402.91, 404.01, 404.03, 404.11, 404.13, 404.91, 404.93, 425.4–425.9, 428.x	I50.2x, I50.3x, I50.4x, I50.84, I50.89, I50.9
Dementia	290.x, 294.1, 331.2	F01.x, F02.x, F03.x, G31.x
Peripheral vascular disease	093.0, 437.3, 440.x, 441.x, 443.1–443.9, 47.1, 557.1, 557.9, V43.4	E75.21, I73.x, I79.8, T82.856, Z95.82x, Z98.62
